# Editorial: The Multiple Facets of Kisspeptin Activity in Biological Systems

**DOI:** 10.3389/fendo.2018.00727

**Published:** 2018-12-03

**Authors:** Rosanna Chianese, William H. Colledge, Silvia Fasano, Rosaria Meccariello

**Affiliations:** ^1^Department of Experimental Medicine, University of Campania L. Vanvitelli, Naples, Italy; ^2^Department of Physiology, Development and Neuroscience, University of Cambridge, Cambridge, United Kingdom; ^3^Department of Movement Sciences and Wellbeing, Parthenope University of Naples, Naples, Italy

**Keywords:** Kisspeptin, cancer, reproduction, puberty, metabolism, comparative genomics, non-hypothalamic activities

In 1996, as part of a screen for anti-metastatic genes, a novel gene termed *Kiss1* was found to be expressed in non-metastatic melanoma cell lines (1). Its 54 amino-acid product, Kisspeptin-54 (Kp-54), was originally called metastin for its ability to inhibit cancer metastasis through the activation of a G protein coupled receptor, previously known as GPR54, and currently renamed the Kisspeptin receptor (KISS1R) (2, 3).

Kp-54 is the longest cleavage product of the Kisspeptin precursor protein, but there are shorter active peptides [i.e., Kp-14, Kp-13, and Kp-10], all capable of binding to KISS1R (3). Since the tissue distribution of KISS1R was very similar in mammalian and non-mammalian vertebrates, especially in the brain, it was suggested that kisspeptins not only acted as metastasis suppressors, but also were a new family of evolutionarily conserved biological modulators (4).

In both rodents and humans, genetic ablation or inactivating mutations of the *Kiss1*/*Kiss1R* genes cause lack of sexual maturation and hypogonadotropic hypogonadism. Conversely, functionally activating mutations of *Kiss1*/*Kiss1R* genes cause precocious puberty (5, 6). Thus, most studies have focused on the involvement of Kisspeptin activity in the central control of reproduction, through the regulation of hypothalamic Gonadotropin Releasing Hormone (GnRH) neurons, which depends on the hormonal milieu, energy homeostasis, and environmental factors (7).

The twelve articles in this Research Topic provide a comprehensive insight into the wider physiological actions of Kisspeptin beyond the reproductive system including cancer, metabolism and neuroscience and also highlight the role of Kisspeptins in non-mammalian species.

Two mini reviews and a review article focus on Kisspeptin and cancer suggesting that the Kisspeptin system is a potential therapeutic agent and/or a prognostic marker for some types of cancer. Ciaramella et al. summarize the latest data concerning the role of Kisspeptin signaling in the suppression of metastasis in cancer, whereas Fratangelo et al. point out additional Kisspeptin activity in tumors especially those with drug resistance. Indeed, new evidence reveals that Kisspeptins can exhibit dual roles in cancer either acting as suppressors of tumorigenesis and metastasis in many cancers or as enhancers in others. In this respect, Guzman et al. highlight the importance of studying cancer in context with attention toward the micro-environment and the steroid receptor status of the cancer cell.

Moving toward the hypothalamic activity of Kisspeptin, the review submitted by Terasawa et al. concerns the contribution of Kisspeptin and Neurokinin B (NKB) signaling in the pubertal increase in GnRH release in female non-human primates. Reciprocal signaling pathways between Kisspeptin and NKB neurons are indispensable to facilitate the pubertal increase in GnRH necessary for the reproductive function in females. In contrast, Kisspeptin as the gatekeeper of reproduction and sex maturation in non-mammalian vertebrates which exhibit more complex GnRH and Kisspeptin systems with multiple forms of ligands and receptors (4, 8), is not as clear as in mammals. In this respect, Ohga et al. summarize Kisspeptin studies in the teleost chub mackerel and elucidate the possible role in fish reproduction and gonadal development. Furthermore, the research article by Pasquier et al. concerns the cloning, sequencing, and differential expression of *Kiss1* and *Kiss2* in both the brain and peripheral tissues from the European eel. A dual inhibitory effect of homologous Kisspeptins on the expression rate of both pituitary LHβ and GnRH receptor2 has been also reported.

A large body of data has established that KISS1 neurons are capable of integrating information about the hormonal milieu, environmental factors, stress signals, metabolism and energy balance, and conveying this information to GnRH secreting neurons. Consequently, the focus on the central role of the Kisspeptins has led to neglecting their possible activities in peripheral tissues. Increasing data reveal that Kisspeptins and KISS1R have a wider expression and possibly a broader spectrum of action in several peripheral tissues such as the gonads, adipose tissue, and liver with direct consequences on gamete quality and fertility rate, pregnancy, energy homeostasis, and body weight control (9).

These points have been fully assessed in this Research Topic. The intricate neuronal networks and the related environmental factors capable to modulate the reproductive axis via KISS1 neurons have been summarized by Yeo and Colledge. These authors also describe the main experimental approaches for investigating functional inputs to KISS1 neurons in the arcuate nucleus. Furthermore, the regulation of the hypothalamic Kisspeptin-KISS1R signaling by metabolic cues and in other situations of energy imbalance like diabetes and obesity has been extensively analyzed by Wahab et al. The direct impact of Kisspeptin on peripheral metabolic tissues has only recently been recognized and emerging data from animal models and clinical studies have been summarized in two review articles. The first one, by Wolfe and Hussain, focuses on the endocrine role of Kisspeptin in the central and local regulation of metabolic functions; the participation of liver-derived Kisspeptin in islet hormone cross-talk and the peripheral sources of circulating Kisspeptin (i.e., placenta and adipose tissues). The second one by Dudek et al. focuses on the local activity of Kisspeptin in peripheral organs such as the pancreas, liver and adipose tissue and the dysregulation of the Kisspeptin system in metabolic diseases (e.g., obesity and diabetes) in humans, situations that are often linked to reproductive disorders and infertility.

Finally, this Research Topic closes with a focus on extra-hypothalamic activity of Kisspeptin in both mammalian and non-mammalian vertebrates. In particular, the review by Ogawa and Parhar provides a survey of Kisspeptin signaling within the habenula of fish, the brain area involved in the neuromodulatory processes of emotional and goal-directed behaviors. Lastly, Stephens and Kauffman summarize what is currently known about the regulation, development, neural projections, and potential functions of Kisspeptin neurons located within the medial amygdala and discuss both the related signaling and the possible implication in many diverse functions and behavioral processes.

Taken together, this Research Topic fills several gaps in Kisspeptin knowledge and provides exciting tools for future directions devoted to the use of Kisspeptin as prognostic/diagnostic biomarkers and therapeutic target for the treatment of cancer and other human diseases like infertility in both sexes and metabolic disorders.

## Author contributions

All authors listed have made a substantial, direct and intellectual contribution to the work, and approved it for publication.

### Conflict of interest statement

The authors declare that the research was conducted in the absence of any commercial or financial relationships that could be construed as a potential conflict of interest.
